# The impact of modulator therapy on the *Pseudomonas aeruginosa* cystic fibrosis lung transcriptome

**DOI:** 10.1073/pnas.2622466123

**Published:** 2026-10-15

**Authors:** Morgana Letizia, Lauren E. Whiteley, Frances L. Diggle, Angela N. Davis, Sarah J. Morgan, Brenda Grogan, John Baugh, Edward F. McKone, Pradeep K. Singh, Marvin Whiteley

**Affiliations:** ^a^ School of Biological Sciences and Center for Microbial Dynamics and Infection, Georgia Institute of Technology, Atlanta, GA 30332; ^b^Emory-Children’s Cystic Fibrosis Center, Atlanta, GA 30322; ^c^ Department of Microbiology, University of Washington School of Medicine, Seattle, WA 98195; ^d^ Department of Medicine, University of Washington School of Medicine, Seattle, WA 98195; ^e^ National Referral Center for Adult Cystic Fibrosis, National Referral Center for Adult Cystic Fibrosis, St. Vincent’s University Hospital, Dublin D04 T6F4, Ireland; ^f^ University College Dublin School of Medicine, Dublin D04 C7X2, Ireland

**Keywords:** *Pseudomonas aeruginosa*, model system, transcriptomics, cystic fibrosis, CF modulators

## Abstract

Chronic lung infection with *Pseudomonas aeruginosa* is a major complication of cystic fibrosis (CF), contributing to lung function decline and increased morbidity and mortality. Laboratory models are used to study CF infections, but their relevance depends on how accurately they reproduce microbial physiology during human infection. We previously developed a framework that uses bacterial gene expression in human infections to quantify model accuracy. Here, we applied this framework to determine how a major therapeutic intervention impacts *P. aeruginosa* physiology in the lungs of people with CF (pwCF) and to develop and evaluate refined preclinical models. We focused on elexacaftor/tezacaftor/ivacaftor (ETI), a highly effective CFTR modulator therapy that has improved outcomes for pwCF. However, evidence indicates that *P. aeruginosa* lung infections persist despite ETI therapy. Analysis of *P. aeruginosa* transcriptomes from human sputum collected before and after ETI initiation revealed minimal changes in gene expression, indicating that the CF airway continues to support a similar bacterial transcriptional state despite therapy. We then evaluated how strain selection, zinc limitation, and polymicrobial coculture influence the ability of laboratory models to recapitulate *P. aeruginosa* gene expression in human CF sputum. Growth of CF clinical isolates in zinc-limited synthetic CF sputum medium produced the most accurate model, whereas population-based cultures and coculture with *Staphylococcus aureus* had limited effects. These findings show that ETI therapy has little impact on the conserved *P. aeruginosa* transcriptional state in human CF sputum and that refined sputum-like models remain useful for studying persistent infection in the postmodulator era.

## Significance

Persistent airway infection by *Pseudomonas aeruginosa* remains a major challenge in cystic fibrosis despite widespread use of highly effective CFTR modulator therapy. Here, we show that *P. aeruginosa* gene expression in the lungs of people with CF remains largely conserved following modulator therapy. Using a transcriptome-based quantitative framework, we leveraged *P. aeruginosa* gene expression from human CF sputum to develop improved preclinical models. The most accurate model was obtained by growing *P. aeruginosa* clinical isolates in zinc-limited synthetic CF sputum medium. These findings establish refined sputum-like systems as useful models for investigating persistent *P. aeruginosa* infection physiology and evaluating future therapeutic strategies in the postmodulator era.

Chronic lung infection with *Pseudomonas aeruginosa* is a major complication of cystic fibrosis (CF) and contributes to progressive loss of lung function and increased morbidity and mortality (1–3). CF is a genetic disease caused by dysfunction of the CF transmembrane conductance regulator (CFTR) protein, a chloride and bicarbonate channel (1). Impaired CFTR activity leads to airway inflammation early in life and the accumulation of viscous mucus (sputum) in the lungs and chronic bacterial colonization. Within this environment, *P. aeruginosa* experiences nutrient conditions and host-derived stress that shape its physiology. Defining this in vivo physiological state is therefore essential for understanding CF infection biology and for developing experimental models that accurately reflect human disease.

Laboratory models are central to CF infection research because they provide experimentally tractable systems for studying bacterial physiology, antimicrobial resistance, and host–pathogen interactions. These models range from simple in vitro cultures and cell-based systems to organoids and animal models, each intuitively designed to capture selected features of the natural infection environment. However, the value of any infection model depends on whether it reproduces the microbial traits observed during human infection, rather than only the physical or compositional features. To address this challenge, we previously established and applied a quantitative framework, termed the accuracy score, that uses bacterial gene expression as a proxy for microbial function to identify the strengths and limitations of model systems and guide improvements in how well they reflect human infection (4–8). This approach directly compares bacterial gene expression in laboratory models with that observed in human infection samples, providing a quantitative measure of how well experimental models reproduce pathogen gene expression in vivo.

Several variables that may shape *P. aeruginosa* function in the CF lung are often considered when developing laboratory models, including strain selection and microbial community context. One debate is whether to use *P. aeruginosa* laboratory reference strains, such as PAO1 and PA14, or clinical isolates that better represent the genomic and phenotypic diversity observed in CF infections (9–14). A second consideration is whether *P. aeruginosa* should be studied alone or in polymicrobial communities. CF lung infections are typically polymicrobial with multiple pathogenic species isolated from expectorated sputum specimens. While the extent of the microbial interactions in the CF lung are not clear, in vitro studies have revealed significant interactions between *P. aeruginosa* and other members of the CF sputum microbiome, particularly *Staphylococcus aureus* (15–19).

The widespread use of highly effective CFTR modulator therapies has introduced a new challenge for CF infection modeling. Elexacaftor/Tezacaftor/Ivacaftor (ETI) triple therapy enhances the function of many mutant CFTRs, improving mucus clearance, airway surface liquid pH, respiratory symptoms, and lung function (20–22). While ETI therapy is also associated with reduced airway pathogen density in people with CF (pwCF), *P. aeruginosa* often persists (22) and may acquire adaptive mutations following ETI therapy (21, 23–25). In addition, evidence from airway lavage samples showed an overall decrease in mucus levels, inflammation, and levels of *P. aeruginosa*-preferred nutrients, including proline, arginine, and lactate, after 1.5 y of ETI therapy, although participants who remained *P. aeruginosa*–infected had higher baseline levels of these factors than those who cleared *P. aeruginosa* (25). Another study experimentally modeled the effect of the biochemical changes in airway mucus associated with ETI on the CF lung microbiome, resulting in altered microbial physiology, competition, and virulence (26, 27). Together, these observations raise the possibility that the post-ETI CF lung represents an altered infection environment in which persistent *P. aeruginosa* may adopt a physiology distinct from that observed before modulator therapy. If so, models optimized against pre-ETI sputum may no longer accurately capture the bacterial state present in contemporary human CF infection.

In this study, we evaluated whether ETI alters *P. aeruginosa* physiology in pwCF by analyzing bacterial transcriptomes, from CF sputum collected before and after ETI therapy. Our approach uses population-level bacterial gene expression as a proxy for microbial function and physiology. We then used the accuracy score framework to determine how strain selection and microbe–microbe interactions influence the ability of laboratory models to reproduce *P. aeruginosa* gene expression in human CF sputum. Finally, we used the post-ETI human transcriptomes to evaluate whether the refined models developed here remain accurate in the current therapeutic era. We found minimal changes in *P. aeruginosa* gene expression in pwCF after ETI administration, indicating that *P. aeruginosa* retains a previously described human infection transcriptional signature despite modulator therapy (4). We also found that clinical isolates grown in a zinc-limited synthetic CF sputum medium most accurately reproduce *P. aeruginosa* gene expression in CF sputum, whereas coculture with *S. aureus* had little impact. Together, these findings show that model accuracy can be improved by incorporating CF-relevant environmental and strain features, and that these refined models remain valuable for studying *P. aeruginosa* physiology in the CF lung in the post-ETI era.

## Results

### Expanded Pre-ETI Human Sputum Transcriptomes Provide a Benchmark for ETI and Model Comparisons.

Previous studies defined a conserved *P. aeruginosa* transcriptional signature in pre-ETI CF sputum samples collected from clinics in Atlanta, GA, and Copenhagen, Denmark (4), and used these data to evaluate the relevance of CF infection models (5, 7). Here, we expanded this pre-ETI dataset with CF sputum samples from Dublin, Ireland, which included samples from individuals not previously on modulator therapy or that had limited clinical response to previous modulator therapies, along with matched post-ETI collections. In total, we analyzed 34 pre-ETI bacterial metatranscriptomes, including 10 newly obtained from Ireland (Dataset S1*A*). All samples were collected before initiation of ETI therapy and immediately preserved after expectoration to maintain RNA integrity.

Metatranscriptomic reads were mapped to the *P. aeruginosa* PAO1 genome as an initial assessment of sample quality and *P. aeruginosa* transcript abundance. PAO1 was used for this first-pass analysis because it is a primary reference strain for genetic and functional studies of *P. aeruginosa* (10), belongs to Group A of the phylogenetic tree alongside the majority of CF isolates (28), contains much of the conserved core genome shared across *P. aeruginosa* isolates (29, 30), and it is the primary strain used in our model systems. This initial mapping allowed us to determine whether each sputum metatranscriptome contained sufficient *P. aeruginosa* reads and gene coverage for downstream analyses. The metatranscriptomes map with high specificity and high read coverage to *P. aeruginosa* PAO1 (*SI Appendix*, Fig. S1). Among the 34 pre-ETI metatranscriptomes, between 1.6 × 10^5^ and 2.9 × 10^6^ reads mapped to the 5,586 *P. aeruginosa* PAO1 protein-coding genes, with only one sample exceeding 1.0 × 10^7^ reads, providing sufficient depth for high-resolution expression profiling across samples. Of the 5,586 protein-coding genes in *P. aeruginosa* PAO1, reads mapped to between 4,628 and 5,484 PAO1 genes across samples (*SI Appendix*, Fig. S1).

To restrict analyses to genes consistently present and expressed in CF-associated *P. aeruginosa* populations, we recalculated the core genome previously defined by Lewin et al. (7) using the expanded dataset. Genes were included if they were present in at least 95% of the 291 complete *P. aeruginosa* genomes, and/or detected in at least 95% of the 34 CF sputum metatranscriptomes. This restriction ensured that downstream comparisons were performed on genes with sufficient representation across samples to support robust statistical analysis. This analysis resulted in an updated core set of 5,168 *P. aeruginosa* genes, which included 21 additional genes compared to the previous core set and was used for downstream accuracy score and differential expression analyses (Dataset S1*B*). To assess whether this framework missed broadly expressed accessory genes, we also examined gene detection across a *P. aeruginosa* pangenome. This analysis showed that the core-gene framework captures the genes with sufficient and consistent read coverage for downstream analysis, whereas most remaining accessory genes were largely isolate- and patient-specific (*SI Appendix*, Fig. S2).

We next used the established accuracy score framework (5, 7, 8) to assess variation in *P. aeruginosa* gene expression across the expanded pre-ETI sputum dataset. This framework is based on a direct comparison of the mRNA levels of each microbial gene in the natural environment (e.g., pre-ETI CF sputum) and in a laboratory model. The first step involves calculating the mean and SD of normalized read counts for each gene in the natural environment, such as the CF lung. Next, the average expression of each gene in a model is compared with the mean and SD of that gene’s expression in the natural environment to determine a z-score. This z-score represents how many SDs the gene’s expression in the model differs from its mean expression in the natural environment. The absolute value of each gene’s z-score is used as a measure of expression similarity between the model and the natural environment, with lower values indicating more similar expression. We define a model’s accuracy score (AS_2_) as the percentage of a microbe’s genes that are expressed within two SDs of that in the natural environment.

In addition to assessing laboratory models, this approach can be used to measure similarity among transcriptomes from the natural environment. To do this, we randomly selected groups of three pre-ETI sputum metatranscriptomes, treated each group as a “model,” and compared their gene expression to that of the remaining 31 sputum metatranscriptomes. This analysis was repeated 3,500 times, yielding a mean AS_2_ of 99.5% (*SI Appendix*, Fig. S1*B*). Thus, the expanded dataset preserves the highly conserved *P. aeruginosa* gene expression profile previously observed in human CF sputum (5, 7) and provides robust benchmark human infection data for subsequent comparisons.

### *P. aeruginosa* Retains a Pre-ETI-Like Transcriptome Following ETI therapy.

**Fig. 1.**
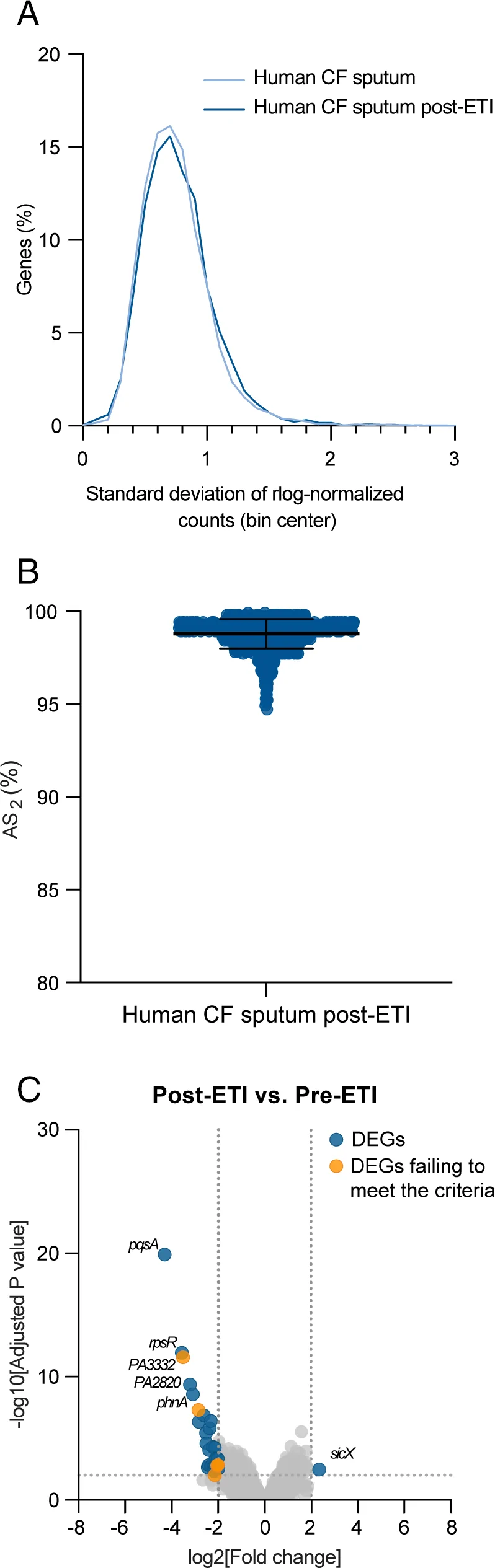
*P. aeruginosa* retains a pre-ETI-like transcriptome following ETI therapy. (*A*) Histogram of the variation in gene expression (core genes, *n* = 5,147) across human CF sputum (pre-ETI) samples and post-ETI samples. Histogram was constructed with a bin size of 0.1 using the variation of rlog-normalized bacterial gene expression levels across metatranscriptomes (human CF sputum pre-ETI, *n* = 34; post-ETI, *n* = 20). (*B*) Accuracy score (AS_2_) for subgroups of three randomly chose post-ETI samples at a time compared to gene expression in the 34 benchmark pre-ETI sputum metatranscriptomes (*n* = 1,140 replicates, exhausting every possible combination of three samples). (*C*) Differential gene expression of *P. aeruginosa* between pre-ETI (*n* = 34) and post-ETI (*n* = 20) transcriptomes. Dashed gray lines indicate the cutoffs (adjusted *P* value < 0.01, |log2[fold change]| > 2) used to identify differentially expressed genes (DEGs). Of the 32 DEGs, 27 genes (in dark blue) had at least one read in ≥75% of the pre- and post-ETI transcriptomes, whereas 5 genes (in orange) did not meet this criterion.

ETI is among the most effective CFTR modulators currently available as it durably increases lung function (31), markedly reduces pathogen burden (21) and lung inflammation (32), and improves CF symptoms (31). However, in most patients, the CF lung remains persistently infected with the same pathogens present prior to treatment (22–25). To evaluate the effect of ETI therapy on *P. aeruginosa* gene expression in humans, we analyzed 20 bacterial metatranscriptomes obtained from expectorated sputum samples collected from adult CF patients receiving ETI for at least 1 y in Dublin, Ireland, and Atlanta, GA (Demographic and clinical characteristics are included in Dataset S1*A* and *SI Appendix*, Tables S1 and S2). Of these, 10 post-ETI samples, collected during Year 1 and/or Year 2 of ETI therapy, were obtained from six of the 10 patients in the Ireland cohort, with matched pre-ETI samples available. Because ETI could alter not only mean gene expression but also the degree of transcriptional variability among patients, we first compared the variance in *P. aeruginosa* gene expression within the pre- and post-ETI sputum datasets. The SD of log-normalized gene expression was calculated for each gene across samples within each group (Dataset S1*C*). The distribution of SD values for post-ETI *P. aeruginosa* gene expression was similar to that observed pre-ETI (Fig. 1*A*), indicating that ETI therapy did not substantially alter the overall level of gene expression variation.

We next compared post-ETI *P. aeruginosa* gene expression to the expanded pre-ETI sputum benchmark using the accuracy score framework. Subgroups of three post-ETI samples were compared with the 34 pre-ETI sputum metatranscriptomes. This analysis was repeated 1,140 times to exhaust every possible three-sample combination. Averaging three samples reduces patient-specific variability and provides a more representative estimate of the shared transcriptional state. This yielded a mean AS_2_ of 98.8% (Fig. 1*B* and Dataset S2*A*), indicating that *P. aeruginosa* gene expression after ETI remains highly similar to the pre-ETI CF sputum transcriptional state.

We further compared *P. aeruginosa* gene expression pre- and post-ETI by performing a differential expression analysis of all 5,168 core genes (Fig. 1*C* and Dataset S2*B*). This analysis identified 32 DEGs, of which 5 did not meet our filtering criterion of having at least one read in 75% of the pre- and post-ETI transcriptomes. This filtering was used to reduce the likelihood that DEGs were driven by strain-specific gene content rather than differences in gene regulation. Among the remaining 27 DEGs, only one gene, *sicX* (33), showed increased expression post-ETI, with a log2 fold change of 2.3. The remaining 26 genes showed decreased expression post-ETI, with only 8 genes exhibiting more than 5.5-fold lower expression relative to pre-ETI levels. The significance and magnitude of these effects were primarily driven by relatively high expression in the Denmark clinic samples and/or high variance among patients (*SI Appendix*, Fig. S3*A*).

Principal component analysis (PCA) of pre- and post-ETI *P. aeruginosa* transcriptomes, restricted to 2,776 genes for which all transcriptomes had at least one mapped read, revealed slightly greater variation among pre-ETI samples across clinics than between pre- and post-ETI samples (*SI Appendix*, Fig. S3*B*). We also repeated differential expression analysis using only paired pre- and post-ETI samples collected from the same six patients at the Dublin clinic (*SI Appendix*, Fig. S3*B* and Dataset S2*C*). No DEGs were identified in this paired analysis. Consistent with this result, PCA of the paired Dublin samples showed that *P. aeruginosa* transcriptomes clustered primarily by patient, with almost no separation between pre- and post-ETI samples except in one patient (*SI Appendix*, Fig. S3*D*). Together, these findings indicate that ETI therapy has minimal impact on the *P. aeruginosa* transcriptome in the CF lung.

### Clinical Isolates and Zinc Limitation Improve Model Accuracy.

Because *P. aeruginosa* retained a highly similar transcriptional state in human sputum after ETI therapy, we next focused on developing highly accurate laboratory models to study this persistent infection state by testing model variables that could best reproduce the conserved CF sputum transcriptome. We previously found that growth of *P. aeruginosa* PAO1 in synthetic CF sputum medium (SCFM2) accurately reproduced the expression of 86% of its genes and that adding the metal-binding host protein complex calprotectin to create a zinc-limited version of this medium increased accuracy to 90% (7). Building on these findings, we used the accuracy score framework to compare *P. aeruginosa* gene expression in models designed to test additional CF-relevant variables, including the use of CF clinical strains and *P. aeruginosa* CF populations. In the clinical strain model, single *P. aeruginosa* isolates recovered from independent sputum samples from pwCF served as biological replicates. In the CF population model, multiple *P. aeruginosa* isolates recovered from the same CF sputum sample were cultured together within each replicate of a model, with the goal of testing whether CF populations containing genotypic/phenotypic diversity improves model accuracy.

**Fig. 2.**
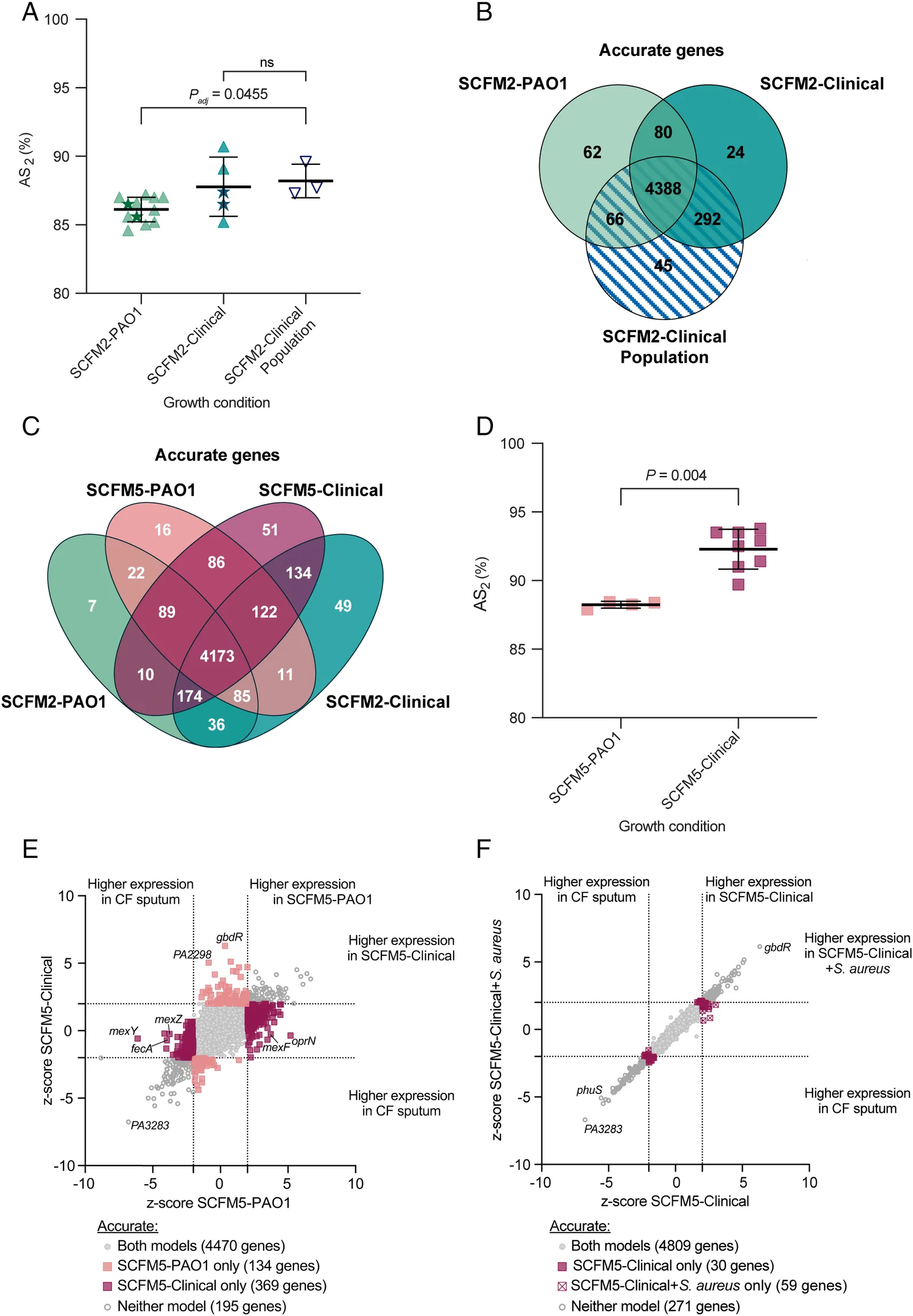
Clinical isolates and zinc limitation improve model accuracy while coculture with *S. aureus* has minimal impact on *P. aeruginosa* model accuracy. (*A*) AS_2_ for each replicate of each model. Significant differences are shown using Kruskal–Wallis and Dunn’s Multiple Comparison Tests. Star symbols in SCFM2-PAO1 and SCFM2-Cinical correspond to the PA14 laboratory strain and LESB58-SED21 clinical isolate grown in SCFM2, respectively. (*B*) Venn diagram showing the shared and unique accurate genes across SCFM2-PAO1, SCFM2-Clinical (single-isolate), and SCFM2-Clinical-Population models. Together, these three models capture the expression of all (4,957) but 211 *P. aeruginosa* PAO1 core genes (4.1%) during CF lung infection. (*C*) Venn diagram showing the shared and unique accurate genes across SCFM2-PAO1, SCFM2-Clinical (single-isolate), SCFM5-PAO1, and SCFM5-Clinical models. Together, these four models capture the expression of all (5,065) but 103 *P. aeruginosa* PAO1 core genes (2%) during CF lung infection. (*D*) AS_2_ for each replicate of each model. Significant differences are shown using the Mann–Whitney test. (*E* and *F*) The average z-score for each gene in each condition is shown, demonstrating how growth of CF clinical isolates in SCFM5 affects the accuracy of PAO1 genes. Coculture with *S. aureus* has a negligible impact on gene expression accuracy. Genes are colored based on the conditions in which they are accurate. Genes are considered accurate when their z-score is between −2 and 2. Annotations are shown for genes of interest.

To determine how model accuracy is influenced by *P. aeruginosa* strains, we grew PAO1 or CF clinical isolates in SCFM2. The CF clinical isolates were grown either as single-isolate cultures or as *P. aeruginosa* populations. Among the CF isolates, we also included LESB58-SED21 (4, 34), which belongs to a highly transmissible *P. aeruginosa* lineage in the United Kingdom, as well as multiple isolates with diverse phenotypes recovered from pwCF in Atlanta, GA (Dataset S1*A*). Growth of *P. aeruginosa* CF isolates in SCFM2 increased model accuracy compared with PAO1, with mean AS_2_ values of 87.8% for single-isolate clinical cultures and 88.2% for population-based clinical cultures (Fig. 2*A* and *SI Appendix*, Fig. S4*A* and Dataset S3*A*). Thus, using CF isolates improved model accuracy, but combining multiple isolates into population-based cultures provided little additional benefit beyond single-isolate clinical cultures. Consistent with this, the accuracy of 316 and 337 genes significantly increased in the single-isolate and population models, respectively, relative to PAO1, with 292 accurate genes shared between the two conditions. The remaining genes exhibited more modest shifts, with z-scores remaining slightly above the twofold threshold (Fig. 2*B* and *SI Appendix*, Fig. S4 *B* and *C* and Dataset S3B). The genes with improved accuracy included several that are commonly mutated or associated with common phenotypes in CF clinical isolates, including genes involved in mucoidy and O-antigen biosynthesis. Interestingly, many genes of unknown function, as well as genes not typically associated with differential expression in CF isolates, also shifted toward sputum-like expression. Overall, these results indicate that using CF isolates improves model accuracy through both expected infection-associated pathways and less well-characterized transcriptional changes, whereas using diverse *P. aeruginosa* populations had only a modest effect under these conditions.

Previous studies from our lab showed that addition of calprotectin enhanced the accuracy of PAO1 grown in SCFM2, likely through restriction of zinc availability (7) (*SI Appendix*, Fig. S5*A*). However, the cost and limited availability of recombinant calprotectin make it difficult to use routinely in laboratory infection models. We therefore tested a zinc-limited variant of SCFM2, called SCFM5, in which zinc availability was restricted by supplementing SCFM2 with 40 µg/mL N,N,N′,N′-tetrakis(2-pyridylmethyl)ethylenediamine (TPEN), a membrane-permeable high-affinity chelator for transition metals and a more practical alternative to calprotectin. Addition of TPEN increased the accuracy of PAO1 grown in SCFM2 from 86.1% to 88.2% (*SI Appendix*, Fig. S5 *A* and *B* and Dataset S3*A*), improving the expression of 235 genes (*SI Appendix*, Fig. S5 *C* and *D* and Dataset S3*B*). TPEN-mediated metal limitation recapitulated much of the calprotectin-associated improvement in model accuracy, although the two conditions were not identical, consistent with differences between a host-derived metal-binding protein and a membrane-permeable chelator. Many genes improved by TPEN are involved in cation binding and transport, including those encoding the Zn^2+^ uptake regulator Zur, ZnuBC high-affinity zinc transporter components, and four other putative zinc uptake systems identified in *P. aeruginosa*: *hmtA* (PA2435), the pseudopaline CntOLMI metallophore pathway (PA4834-PA4838), PA2911-PA2914, and PA4063-PA4066. Also accurately expressed were the zinc-independent paralogs of the 50S ribosomal proteins L31 (*rpmE2*) and L36 (*rpmJ2*) (PA3600-PA3601), which lack structural zinc-binding sites, as well as the alternative regulator DksA2, which is involved in the stringent response under zinc-limiting conditions (*SI Appendix*, Fig. S5*D*). Notably, the expression of 133 genes among the 316 genes accurately recapitulated in the SCFM2-Clinical model was also accurately reproduced when PAO1 was grown in SCFM5, indicating that TPEN-mediated metal limitation is sufficient to restore human sputum-like expression for this subset of genes in a laboratory strain (Fig. 2*C* and Dataset S3*B*).

The highest model accuracy was achieved when *P. aeruginosa* clinical isolates, with diverse phenotypes recovered from pwCF in Atlanta, GA (Dataset S1*A*), were grown in SCFM5. In this condition, 369 genes showed expression levels that more accurately captured those observed in CF sputum compared to PAO1, resulting in an AS_2_ of 92.3% for the SCFM5-Clinical model (Fig. 2 *C* and *D* and *SI Appendix*, Fig. S5*E* and Dataset S3*A*). Interestingly, 142 of these genes that had z-scores higher than |2.5| when PAO1 was grown in SCFM5 shifted toward sputum-like expression levels, with their z-scores falling within 2 SD of their mean expression in CF sputum (Fig. 2*E* and Dataset S3*B*). These genes are mainly involved in iron acquisition, amino acid metabolism, osmotic and oxidative stress responses, motility, and antibiotic resistance. The remaining 227 genes showed more modest shifts, with z-scores already approaching the twofold threshold in PAO1. Together, these findings indicate that clinical CF isolates more closely reflect in vivo-like behavior under SCFM5 conditions than PAO1, consistent with adaptation to nutrient limitation, host-derived stress, and chronic infection in the CF airway. Notably, a core set of 134 genes was consistently accurate across both SCFM2-Clinical and SCFM5-Clinical models, further supporting the idea that clinical isolates are better matched to sputum-like conditions than laboratory strains (Fig. 2*C*).

### Coculture with *S. aureus* Has Minimal Impact on *P. aeruginosa* Model Accuracy.

CF lung infections are commonly polymicrobial, and *S. aureus* is frequently detected with *P. aeruginosa* in airway samples from pwCF. In addition, extensive in vitro work has shown that interactions between these species can alter growth, metabolism, antibiotic tolerance, and spatial organization (15, 16, 35–39). We therefore evaluated whether coculture with *S. aureus* improves the ability of models to reproduce *P. aeruginosa* gene expression in CF sputum. *P. aeruginosa* PAO1 or CF clinical isolates were grown in SCFM5 in the presence or absence of *S. aureus* LAC, and *P. aeruginosa* gene expression was compared to the human sputum benchmark. *P. aeruginosa* and *S. aureus* coexist in this model at bacterial numbers similar to those observed in monoculture growth (15). Coculture with *S. aureus* produced AS_2_ values of 88.3% for SCFM5-PAO1 and 92.0% for SCFM5-Clinical (*SI Appendix*, Fig. S6 *A*–*C* and Dataset S3*A*), values similar to the corresponding *P. aeruginosa* monoculture conditions. The presence of *S. aureus* had minimal impact on *P. aeruginosa* gene expression, with only a few genes showing borderline differences in accuracy between the presence and absence of *S. aureus*, as indicated by z-scores falling slightly outside of 2 (Fig. 2*F* and *SI Appendix*, Fig. S6 *C* and *D* and Dataset S3*B*). Because eight of the 34 sputum samples used to construct the human benchmark were *S. aureus*-positive (Dataset S1*A*), we next asked whether their inclusion influenced the apparent accuracy of the coculture models. Recalculating AS_2_ using only the 26 *S. aureus*-negative sputum transcriptomes yielded nearly identical values of 88.1% for SCFM5-PAO1 + *S. aureus* and 91.9% for SCFM5-Clinical + *S. aureus*. Thus, under the conditions tested, coculture with *S. aureus* did not substantially improve the ability of the model to reproduce the bulk *P. aeruginosa* transcriptome observed in human CF sputum.

### Refined Models Remain Accurate When Benchmarked Against Post-ETI Human Sputum.

**Fig. 3.**
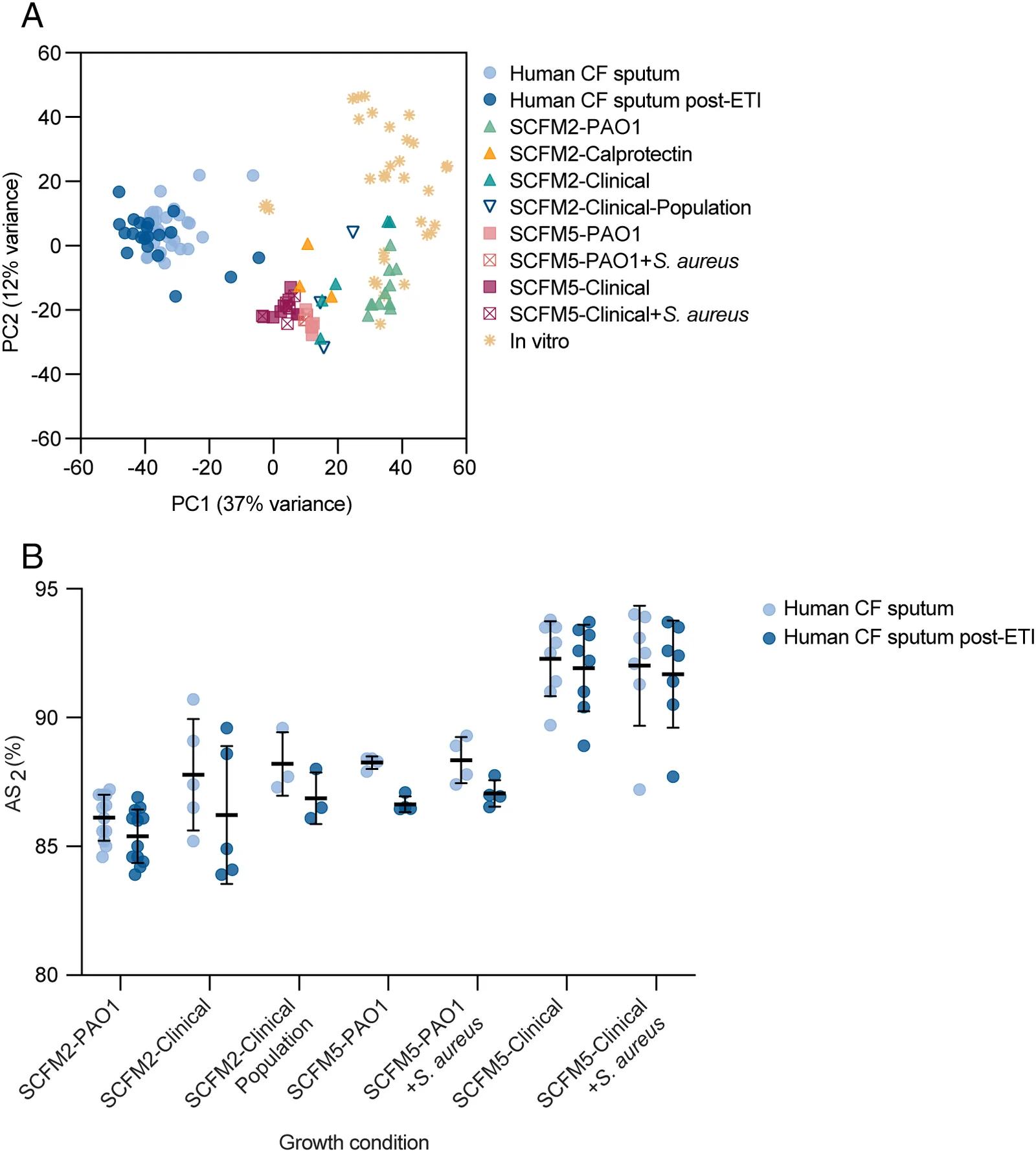
Refined models remain accurate when benchmarked against post-ETI human sputum. (*A*) PCA of *P. aeruginosa* gene expression in pre- and post-ETI human CF sputum samples and preclinical model systems. The PCA was constructed using Variance Stabilizing Transformation (VST)-normalized read counts of the 500 genes (out of 5,168 core genes) with highest variability across samples using DESeq2. The in vitro group includes the 35 standard in vitro laboratory models described in Dataset S1*A*. (*B*) AS_2_ for each replicate of each model compared to pre-ETI (in light blue) and post-ETI (in dark blue) CF sputum transcriptomes. No significant differences were found after analysis with multiple Mann–Whitney test.

Having identified SCFM5-PAO1 and SCFM5-Clinical as the most accurate models relative to pre-ETI human CF sputum, we next asked whether these models remain accurate in the postmodulator era. We first assessed overall relationships among *P. aeruginosa* transcriptomes from pre- and post-ETI CF sputum and several in vitro and in vivo laboratory model systems (Dataset S1*A*). PCA showed that all human CF sputum samples clustered together, with clear separation between human sputum transcriptomes and those of laboratory models (Fig. 3*A* and *SI Appendix*, Fig. S7). This separation occurred primarily along the first principal component, which accounted for approximately 37% of the total variance. Improved models incorporating clinical isolates and zinc-limited SCFM clustered between human sputum transcriptomes and standard in vitro laboratory models (Dataset S1*A*). We then recalculated the accuracy scores of the SCFM5-PAO1 and SCFM5-Clinical models relative to the *P. aeruginosa* transcriptomic profile in post-ETI human CF sputum (Dataset S3*C*). AS_2_ values showed no significant difference compared to those calculated relative to pre-ETI transcriptomes (Fig. 3*B*), indicating that the refined sputum-like models developed here remain accurate when benchmarked against *P. aeruginosa* transcriptomes post-ETI therapy. These findings show that ETI does not substantially alter the conserved *P. aeruginosa* transcriptional state detected in sputum and that current refined models remain suitable for studying persistent *P. aeruginosa* physiology in the post-ETI era.

## Discussion

*P. aeruginosa* gene expression is driven by signals and cues in the local environment. Given the broad effects of ETI on CFTR function, lung physiology, mucus hydration/composition, amino acid levels, and pathogen burden (20, 21, 23–25, 40), one prediction might be that ETI would create a sufficiently altered CF airway environment to change *P. aeruginosa* gene expression. Our data do not support this prediction. Instead, *P. aeruginosa* in human CF sputum after ETI retained a transcriptome that was highly similar to the pre-ETI CF sputum transcriptome. Our data do not exclude the possibility of unique transcriptional changes occurring within individuals; however, across individuals, we did not identify a consistent bulk response of *P. aeruginosa* to ETI and paired pre- and post-ETI transcriptomes generally clustered closely within individuals. While this may initially seem surprising, it is consistent with our previous discovery of a conserved *P. aeruginosa* transcriptional signature during human infection, including in pre-ETI CF sputum, burn wounds, and chronic wounds (4). The *P. aeruginosa* post-ETI transcriptome maintains this broader human infection signature, suggesting that the dominant signals and cues driving *P. aeruginosa* gene expression in the CF lung after ETI are similar to those present in other human infections. This conserved human gene expression signature has relevance beyond CF lung infections, allowing prediction of *P. aeruginosa* physiology and behavior across human infection sites, even when direct transcriptomic data are not available. Of note, the *P. aeruginosa* transcriptomes used to define this human infection signature have come from chronic infections, and in the future, it will be important to determine whether this transcriptional signature is also maintained in acute infections.

While the similarity between pre- and post-ETI transcriptomes most likely reflects persistence of an environment that supports this transcriptional state, rather than an inability of *P. aeruginosa* to respond to environmental change, other explanations remain possible. For example, localized or transient responses to the post-ETI environment could be obscured by bulk transcriptomic measurements. The timing of the post-ETI samples could also have excluded a transient transcriptional response occurring shortly after ETI initiation or potential population bottleneck selection, with bacterial populations subsequently returning to a stable chronic transcriptional state as the post-ETI environment was established. The cohort sampled here may include individuals with persistent mucus retention, which could maintain a local bacterial growth environment more similar to that present before ETI. It is also possible that we may be preferentially sampling bacteria from regions within the lung that do not robustly respond to ETI, and thus the environment remains similar to that found in the pre-ETI lung. Additionally, as our conclusion that the *P. aeruginosa* transcriptome is largely conserved in patients receiving established ETI therapy is based on bulk transcriptomic analyses, we cannot rule out the possibility that specific subpopulations may undergo substantial physiological changes following ETI while contributing relatively little to the overall population average. Finally, it will be important in future studies to reanalyze these data in the context of potential genetic bottlenecks and bacterial adaptation following ETI therapy (23, 24).

The model-comparison experiments reinforce a simple point about model development: Factors that seem likely to be important in a model, such as the presence of particular environmental components, microbial species, or strains, do not necessarily improve how well a model recapitulates a *P. aeruginosa* human infection gene expression pattern. In some cases, these factors do matter, as model accuracy compared to both pre- and post-ETI transcriptomes was highest when CF clinical isolates were grown under zinc-limited SCFM (SCFM5) conditions (AS_2_ of 92%). These data indicate that despite reduction of the zinc-chelating protein complex calprotectin in post-ETI CF sputum (32), *P. aeruginosa* still shows a zinc-limited transcriptomic signature in this environment, similar to that observed pre-ETI and in chronic wounds and burn wounds (4, 5, 7). However, it should be noted that growing the laboratory strain PAO1 in SCFM5 also resulted in a highly accurate model (AS_2_ of 88%), indicating that small changes to an in vitro growth regime can substantially improve the accuracy of models using both clinical and laboratory strains. This is important because much of what is known about *P. aeruginosa* physiology, genetics, and regulation comes from studies using laboratory strains. Our data suggest that, when grown under appropriately refined conditions, laboratory strains can provide valuable insight into the physiology and behavior of *P. aeruginosa* in the CF lung.

By contrast, two other biologically motivated additions, within-sample *P. aeruginosa* populations and coculture with *S. aureus*, had little effect on model accuracy. Within-sample populations were included because multiple *P. aeruginosa* isolates with different genotypes and phenotypes can be recovered from the same CF sputum sample. Thus, it seemed reasonable that including multiple *P. aeruginosa* strains from a single CF sputum sample might better recapitulate gene expression in the CF lung. However, these populations did not substantially improve model accuracy compared with single CF isolates. This does not mean that strain heterogeneity is unimportant, as it may still influence specific phenotypes such as antibiotic tolerance. Rather, for the specific goal of recapitulating the bulk *P. aeruginosa* human infection gene expression pattern, adding multiple isolates provided little additional benefit under the conditions tested. Coculture with *S. aureus* led to a similar conclusion. Although *P. aeruginosa* and *S. aureus* clearly interact in vitro (15–17, 19, 36–38, 41) adding *S. aureus* had little impact on the bulk *P. aeruginosa* transcriptome in SCFM5. Again, this does not rule out polymicrobial interactions in the CF lung. Rather, it suggests that a simple pairwise coculture is not automatically a more accurate model of *P. aeruginosa* physiology in the CF lung. *P. aeruginosa* and *S. aureus* may still interact during CF lung infection, but the interactions may be transient and therefore missed by bulk transcriptomic measurements, or they may occur at the micron scale within a minor portion of the population (15, 19). Thus, population diversity and polymicrobial interactions may matter during CF lung infection, but they did not improve model accuracy in the way that zinc limitation and CF isolate background did.

Overall, this study supports two main conclusions. First, the *P. aeruginosa* population that persists in human CF sputum after ETI retains a conserved CF human infection transcriptional signature. Second, refined sputum-like models, especially those using CF clinical isolates under zinc limitation, remain useful for studying that persistent bacterial state. Thus, the central problem after ETI may not be that *P. aeruginosa* has adopted a new postmodulator physiology, but that the CF lung continues to preserve the signals and cues that drive the persistence of this bacterium.

## Materials and Methods

### CF Sputum Collection.

Expectorated CF sputum was collected in RNAlater from the Saint Vincent’s Hospital CF Clinic in Dublin, Ireland, and the Emory-Children’s Center for Cystic Fibrosis and Airways Disease Research by the Cystic Fibrosis Biospecimen Laboratory as previously described (4). Patients had stable infections and were not undergoing acute exacerbation. Samples were collected before and at the indicated time points following ETI modulator therapy. Most pre-ETI samples from the Dublin cohort were obtained from subjects taking either lumacaftor/ivacaftor or tezacaftor/ivacaftor, along with matched post-ETI collections from a subset of the cohort. Collections were approved by Georgia Tech IRB protocol H18220. Samples were deidentified prior to use in this study.

### *P. aeruginosa* In Vitro Growth Conditions.

*P. aeruginosa* MPAO1 (42), PA14 (43), and indicated clinical isolates were used for in vitro experiments. SCFM1, SCFM2, and SCFM5 media have been purchased from SynthBiome, USA. Cells were grown overnight in SCFM1 medium. Overnight cultures were inoculated into SCFM2 or zinc-limited SCFM5 media at a final OD600 of 0.05 or 0.1 and grown statically at 37 °C. Deep-well 96-well plates had a total of 500 µL of medium per well. After 5 h or 6 h, respectively, cultures from 8 wells per sample were combined and preserved in RNAlater (Thermo Fisher Scientific) for RNA extraction. PAO1 was grown in monoculture or in coculture with *S. aureus* US300 (LAC). The strains and culture conditions are indicated in Dataset S1*A* for each sample.

### RNA Extraction and Sequencing.

RNA was extracted from human CF sputum samples and experimental model systems as previously described in ref. 5. Briefly, samples were thawed, RNAlater was removed, and samples were resuspended in RNase-free TE buffer containing lysozyme and lysostaphin. Samples were incubated for 30 min at 37 °C for enzymatic lysis, RNA Stat-60 (Amsbio) was added, and samples were bead beat three times for 30 s each, placing on ice in between each round. Following lysis, chloroform was added to each sample, samples were mixed and centrifuged to separate phases. The aqueous phase was transferred to a new tube, and the RNA was precipitated with isopropanol. All RNA was treated with DNase I (Promega) in solution, cleaned up with RNA Stat-60 as described above (all steps following the lysis), and quantified using a Qubit fluorometer. When necessary, RNA was fragmented using the NEBNext Magnesium fragmentation module (New England Biosciences) per the manufacturer’s instructions. Sequencing libraries were prepared as described in ref. 7 with the NEBNext Small RNA Library prep kit (New England Biosciences), and adapter dimers were removed by size selection on a 5% TBE polyacrylamide gel. Ribosomal RNAs (rRNAs) were removed using the Invitrogen MICROBExpress Bacterial mRNA Enrichment Kit or the Qiagen QIAseq FastSelect Kits (HMR and 5S/16S/23S), as indicated in Dataset S1*A*, following a modified protocol. Briefly, the QIAseq rRNA depletion was performed after ligating 3′ and 5′ adaptors to RNA fragments, resulting in a reaction volume of 30 µL, while the QIAseq kit was designed for a reaction volume of only 15 µL. To accommodate the full 30 µL starting sample, we adjusted the volume of the FastSelect FH Buffer and used 40 µL beads in the first step of the depletion process (volumes of downstream steps remained the same). Libraries for new samples reported here were sequenced at the Molecular Evolution Core at the Georgia Institute of Technology on an Illumina NextSeq 500 using 75-bp single-end runs.

### *P. aeruginosa* Genome Annotation and Pangenome Analysis.

Core genes were defined as the 5,168 PAO1 genes that were either present in 95% of the 291 complete *P. aeruginosa* genomes selected in ref. 7 and/or expressed in 95% of the 34 human sputum samples included in this study. Hierarchical TIGRFAM annotations from ref. 7 were used to provide hand curated annotations for all PAO1 core genes, with “unknown function” being used in all cases where the annotation was unknown.

### RNA-seq Analysis.

Published RNAseq datasets for CF sputum, standard mouse acute pneumonia model, SCFM2 mouse infection model, epiSCFM2, SCFM2, and other in vitro samples were downloaded from the NCBI Sequence Read Archive (SRA) database (see Dataset S1*A* for accession numbers and references). Published and new RNAseq data were analyzed as described (7). RNA-seq read quality was confirmed with FastQC v0.12.1 (44). Reads were trimmed using Cutadapt v2.6 to remove Illumina adapters from the 3’ end, and reads were retained that were at least 22 base pairs long (45). To remove non–*P. aeruginosa* reads, trimmed reads were mapped with bowtie2 v2.3.5.1 using default parameters to a metagenome of 105 “decoy” strains from 59 species, based on species previously identified in sputum samples (5, 7, 46). The reads that did not map to the decoy genomes were then mapped to *P. aeruginosa* PAO1 (Accession number GCF_000006765.1) using bowtie2. featureCounts v2.0.1 was used to assign mapped reads to PAO1 genes with the flags -s 1 (forward stranded) or -s 2 (reverse stranded) (strandedness information is provided in Dataset S1*A*) and -O (allowMultiOverlap) so that each read was assigned to a single locus or to neighboring genes (47). At each step, MultiQC v1.10 was used to track analysis quality (48).

Read counts for each sample were combined into a counts matrix for use in downstream analyses. RNA-seq analyses were performed using count data for the 5,168 core genes, with variance stabilizing transformation (VST)-normalization performed on all samples together in DESeq2 v1.46.0 with blind = TRUE in R (49).

### Accuracy Score Analyses.

Accuracy scores were calculated following the approach developed and used in refs. 5 and 7. All analysis scripts are available at https://github.com/glew8/PA_ModelAccuracy. Briefly, the mean and SD of expression of VST-normalized count data for the human expectorated CF sputum samples were determined. Then, z-scores and AS_2_ values were calculated for each experimental model system using two approaches. In the first approach, which was used to calculate the overall AS_2_ for each model, z-scores for each gene were calculated for each replicate of an experimental model, the z-scores were rounded to 4 decimal places, and then the AS_2_ values for each replicate were calculated as the percentage of genes with a z-score between −2 and 2. The ROUT method in Prism10 (GraphPad Software, Boston, MA, https://www.graphpad.com”) was used to identify outliers (50), which identified the human CF sputum sample EM24_US as an outlier and was excluded from all further analyses. Then, the overall AS_2_ for the experimental model was calculated using the mean AS_2_ score across replicates. In the second approach, which was used to compare across models at the gene and functional levels (e.g., in Venn Diagrams and TIGRFAM analyses), the z-score of each gene was calculated as the mean z-score across replicates, rounded to four decimal places. Then, the AS_2_ for each functional category was calculated as the percentage of genes with a z-score between −2 and 2. “Sunburst plots” showing the TIGRFAM annotations were constructed using ggsunburst and the scico palette “romaO”(51, 52).

### Gene Expression Analyses.

For PCA and differential expression, DESeq2’s VST function was used with blind = TRUE in R (v1.46.0) (49). PCA of *P. aeruginosa* gene expression in human CF sputum and experimental model systems was performed using the top 500 most variable genes. Data for gene expression variance in Fig. 1*A* were normalized with DESeq2’s rlog function. All figures were created using Prism10 (GraphPad Software, Boston, MA, https://www.graphpad.com).

## Supplementary Material

Appendix 01 (PDF)

Dataset S01 (XLSX)

Dataset S02 (XLSX)

Dataset S03 (XLSX)

## Data Availability

Accession numbers for previously published samples can be found in Dataset S1*A*. RNA-seq reads from the new samples included in this paper were deposited in the NCBI Sequence Read Archive database under BioProject No. PRJNA1474456 (53). For the sputum samples, only reads mapping to *P. aeruginosa* were uploaded. Raw and normalized count data and z-scores are available in Datasets S1 *C*–*E* and S3 along with the hand curated annotation of the *P. aeruginosa* PAO1 genome (Dataset S1*B*). The pangenome data are available in ref. 7. All other data are included in the manuscript and/or supporting information. Previously published data were used for this work [(4–7, 54–57), Dötsch et al., PLoS One, 2012; Dötsch et al., mBio, 2015; Murray et al., mBio, 2015; Fritz et al., APMIS, 2022].
