# Supplementary material for: The impact of modulator therapy on the Pseudomonas aeruginosa cystic fibrosis lung transcriptome
Source: Proc Natl Acad Sci U S A. 2026 Oct 15;123(42):e2622466123. doi: 10.1073/pnas.2622466123 (PMC13684289; doi:10.1073/pnas.2622466123)
Supplement: Supplementary file 1 [file pnas.2622466123.sapp.pdf]

PNAS

Supporting Information for

The impact of modulator therapy on the *Pseudomonas aeruginosa* cystic fibrosis lung transcriptome

Letizia, Morgana; Whiteley, Lauren E.; Diggle, Frances L.; Davis, Angela N.; Morgan, Sarah J.; Grogan, Brenda; Baugh, John; McKone, Edward F.; Singh, Pradeep K.; Whiteley, Marvin

Marvin Whiteley
Email: mwhiteley3@gatech.edu

This PDF file includes:

Figures S1 to S7
Table S1 and S2
Legends for Datasets S1 to S3

Other supporting materials for this manuscript include the following:

Datasets S1 to S3

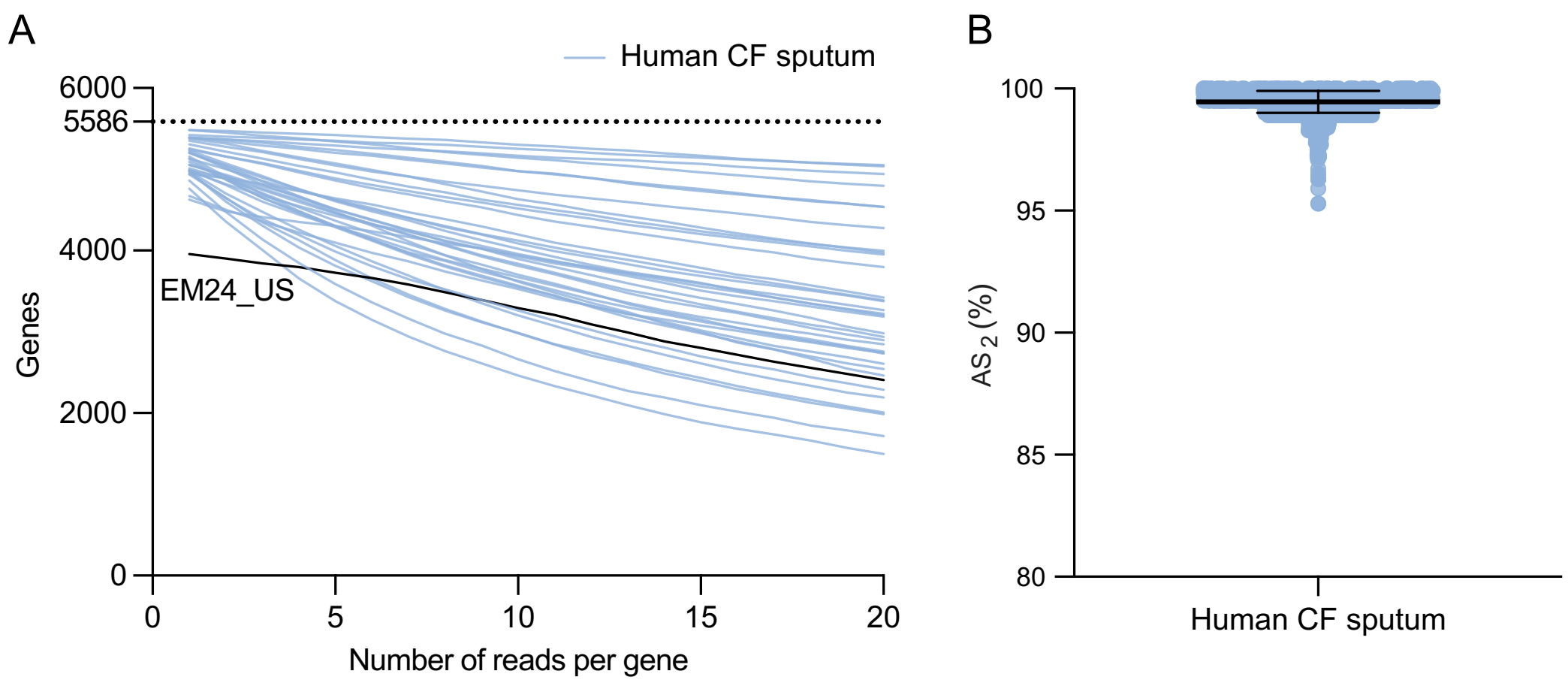

Fig. S1. *P. aeruginosa* gene expression in human CF sputum metatranscriptomes. (*A*) Coverage of the *P. aeruginosa* PAO1 genome. Each line represents the depth of coverage for PAO1 genes for an individual human sputum metatranscriptome, demonstrating sufficient coverage for the 34 metatranscriptomes in light blue used in this study (Human_Sputum_EM24_US in black was identified as an outlier and excluded from all further analyses). The dashed line shows the 5,586 total protein coding genes in the PAO1 genome. (*B*) AS_2 across the 34 pre-ETI *P. aeruginosa* CF sputum transcriptomes by randomly selecting three samples as the "model" and comparing gene expression to the remaining 31 sputum metatranscriptomes (*n* = 3,500 replicates).

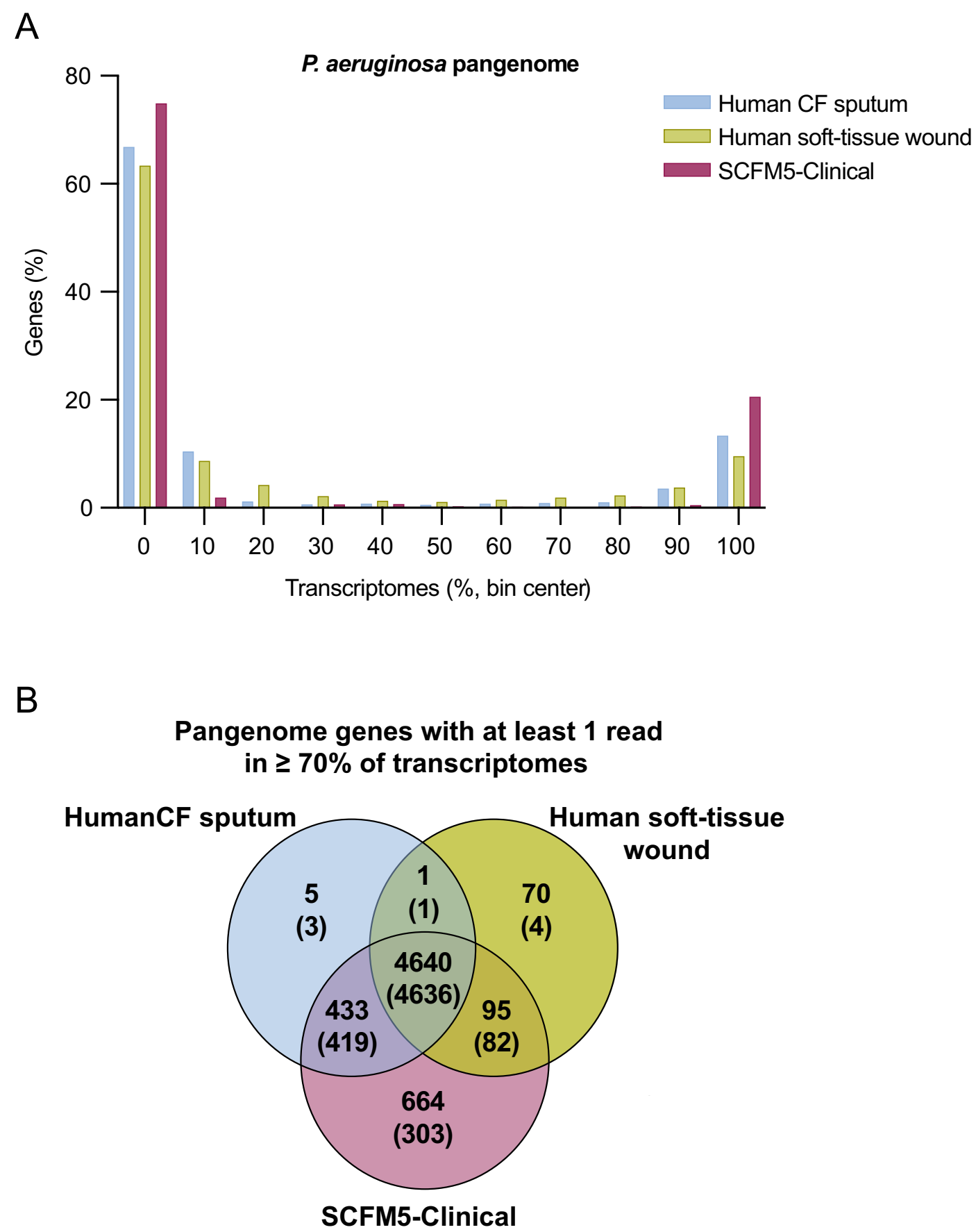

Fig. S2. Prevalence of *P. aeruginosa* pangenome genes in transcriptomes from human samples. (*A*) Percentage of pangenome genes (*n* = 27,352) detected with at least one read in *P. aeruginosa* transcriptomes from human samples across infection sites or models. Histogram was generated using a bin size of 10 based on the percentage of transcriptomes in which each gene was detected within each group (human CF sputum, *n* = 34; soft-tissue wound, *n* = 10; SCFM5-Clinical, *n* = 8). (*B*) Venn diagram showing the shared and unique pangenome genes across human CF sputum, soft-tissue wound and SCFM5-Clinical transcriptomes. Genes belonging to the PAO1 genome are indicated in brackets.

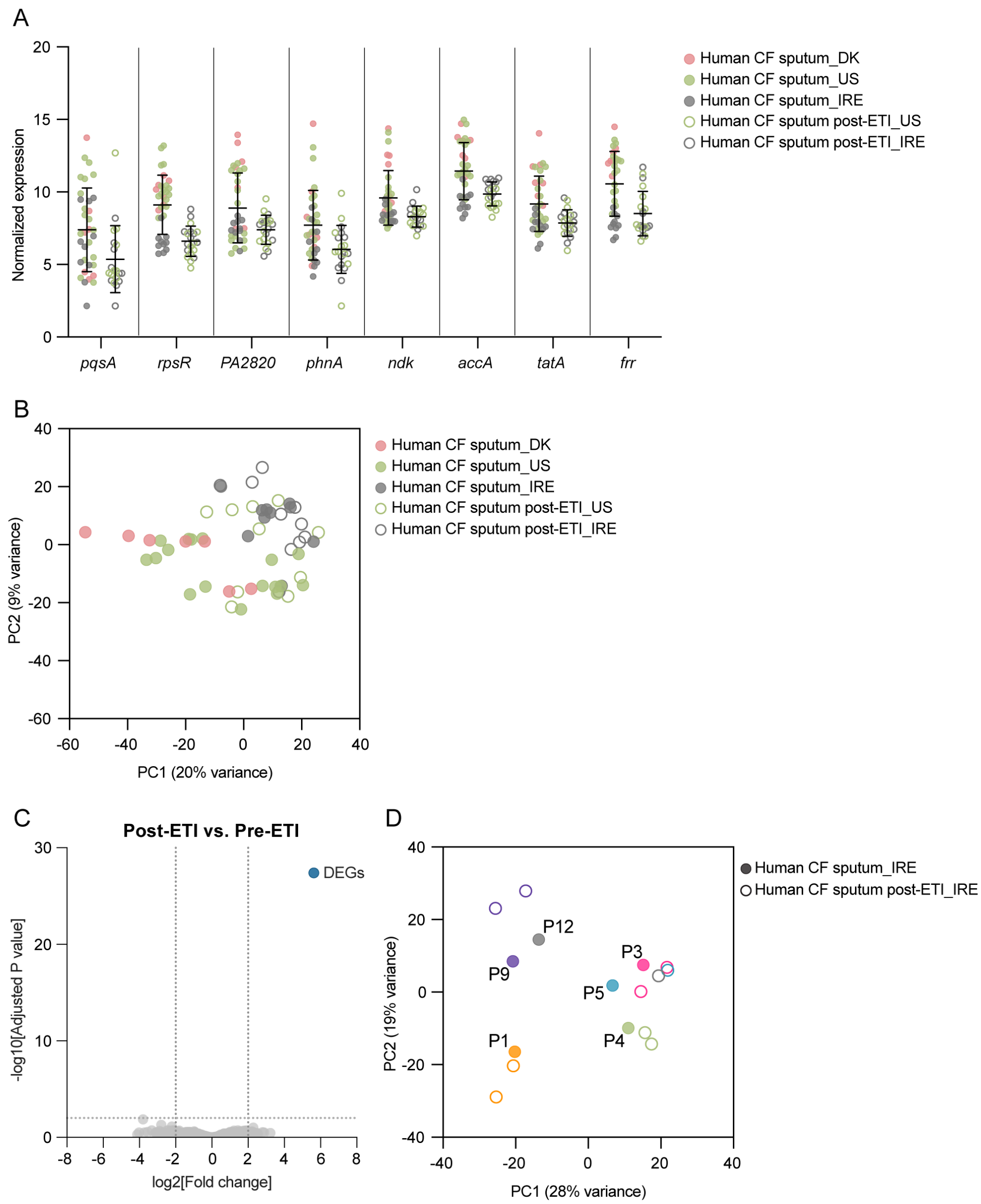

Fig. S3. *P. aeruginosa* retains a pre-ETI-like transcriptome following ETI therapy. (*A*) Mean VST-normalized expression of the 8 *P. aeruginosa* genes exhibiting more than 5.5-fold lower expression post-ETI (open circles) compared with pre-ETI (filled circles) across the pre- and post-ETI samples analyzed. (*B*) Principal component analysis (PCA) of *P. aeruginosa* gene expression in pre- and post-ETI human CF sputum transcriptomes. The PCA was constructed using Variance Stabilizing Transformation (VST)-normalized read counts of the 500 genes (out of 2,776 genes for which all transcriptomes had at least one read mapping) with highest variability across samples using DESeq2. (*C*) Differential gene expression of *P. aeruginosa* between pre-ETI (n = 6) and post-ETI (n = 10) samples collected from the same six patients at the Dublin clinic. Dashed grey lines indicate the cutoffs (adjusted P value < 0.01, |log2[fold change]| > 2) used to identify differentially expressed genes (DEGs). (*D*) PCA of *P. aeruginosa* gene expression in pre- (filled circles) and post-ETI (open circles) human CF sputum samples collected from the same six patients at the Dublin clinic. The PCA was constructed using VST-normalized read counts of the 500 genes (out of 5,168 core genes) with highest variability across samples using DESeq2.

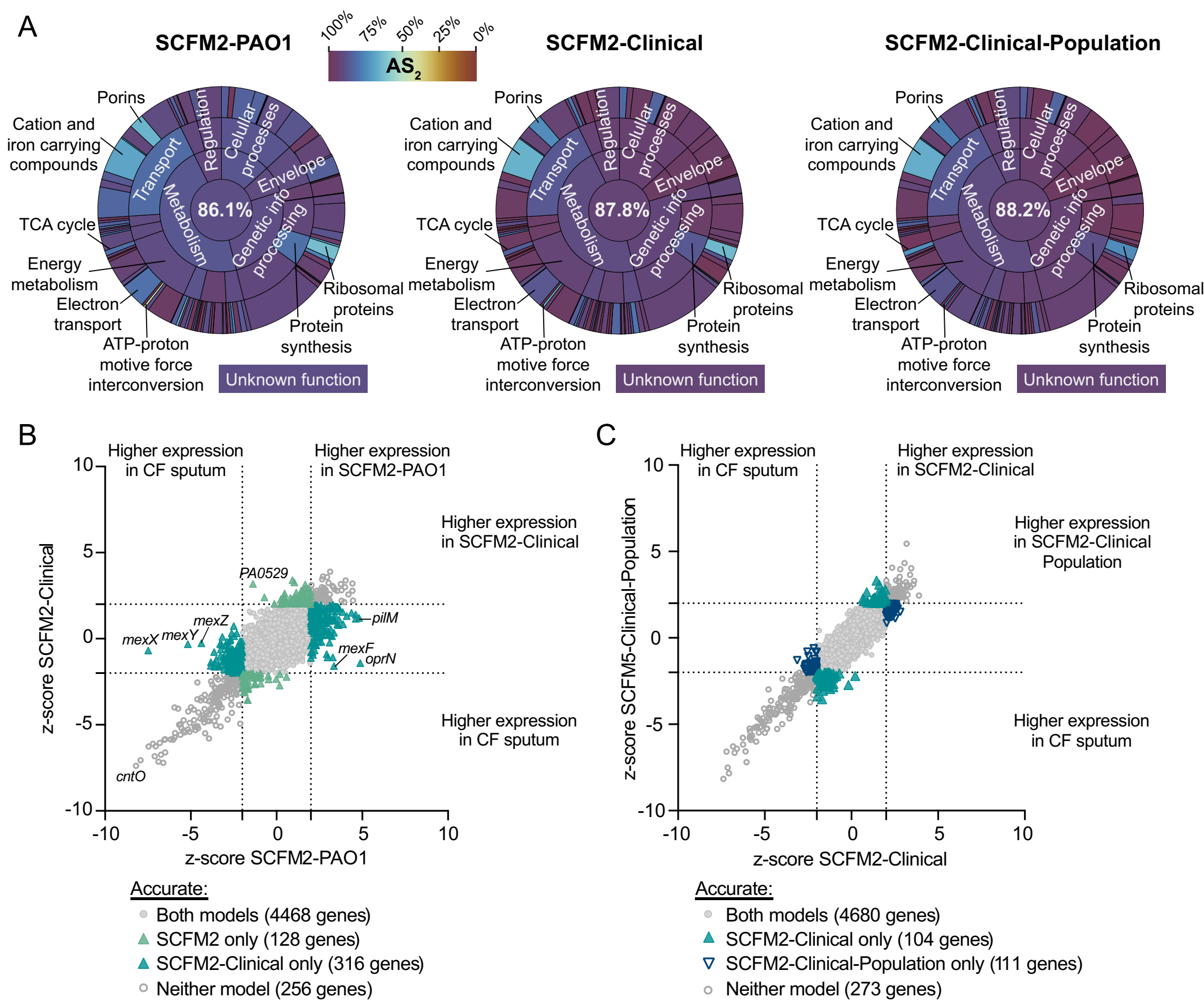

Fig. S4. Using CF isolates improves model accuracy but population-based cultures do not substantially outperform single-isolate cultures. (*A*) AS_2 for TIGRFAM subcategories for *P. aeruginosa* PAO1 and CF clinical strains in SCFM2. The color in the middle represents the average AS_2 across individual replicates for all PAO1 genes (those with and without TIGRFAM designations). The next level out from the middle of the circle contains TIGRFAM meta roles, the next contains TIGRFAM main roles, and the outer-most layer contains TIGRFAM sub roles. The area of each category is proportional to the number of genes in that category. (*B* and *C*) The average z-score for each gene in SCFM2-PAO1, SCFM2-Clinical (single-isolate) and SCFM2-Clinical-Population, demonstrating how the growth of CF clinical isolates in SCFM2 affects the accuracy of PAO1 genes. Genes are considered accurate when their z-score is between −2 and 2. Genes are colored based on the conditions in which they are accurate, and annotations are shown for select genes.

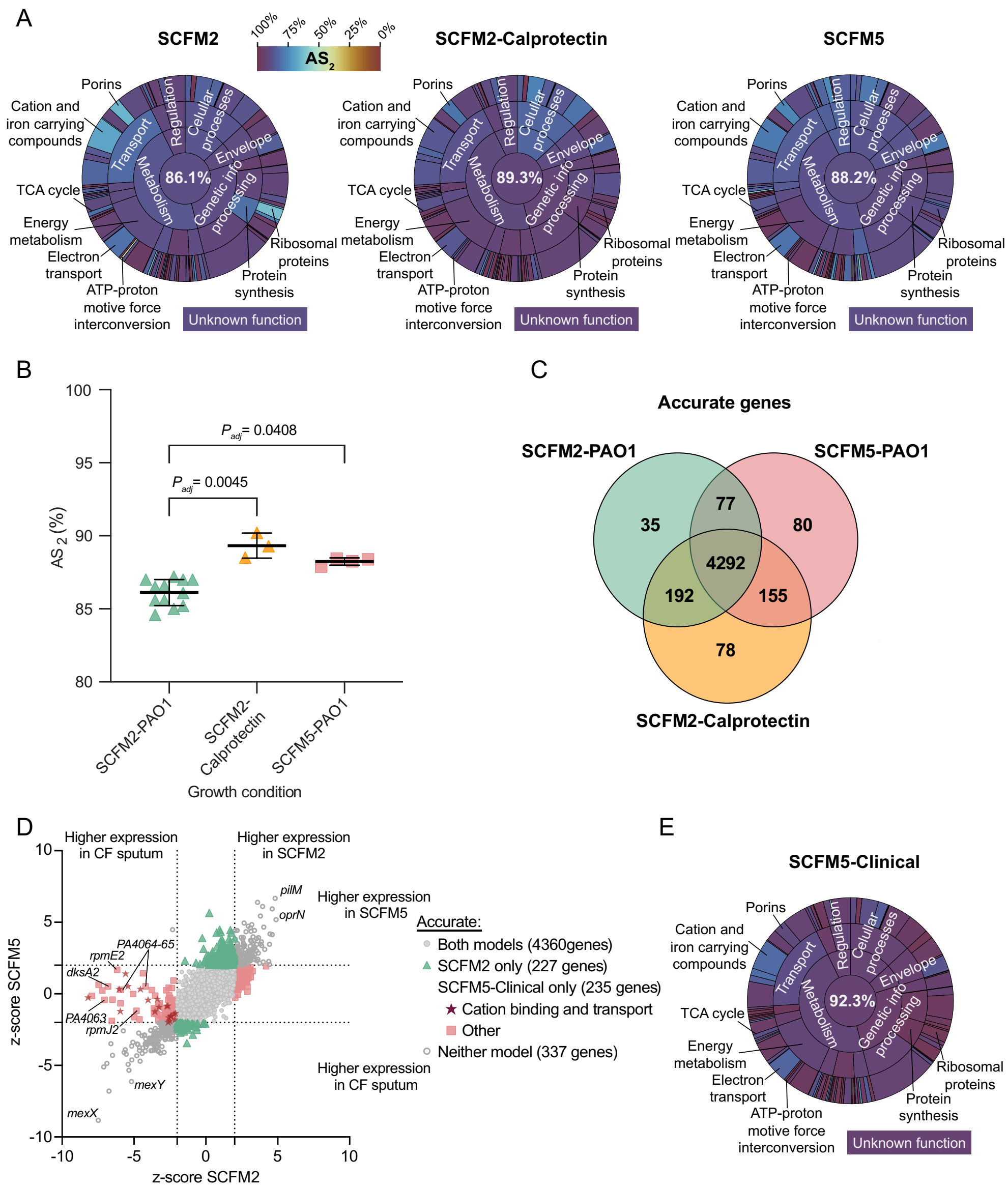

Fig. S5. Clinical isolates and zinc limitation improve model accuracy. (*A*) AS_2 for TIGRFAM subcategories for *P. aeruginosa* PAO1 in SCFM2, SCFM2-Calprotectin and SCFM5. The color in the middle represents the average AS_2 across individual replicates for all PAO1 genes (those with and without TIGRFAM designations). The next level out from the middle of the circle contains TIGRFAM meta roles, the next contains TIGRFAM main roles, and the outer-most layer contains TIGRFAM sub roles. The area of each category is proportional to the number of genes in that category. (*B*) AS_2 for each replicate of each model. Significant differences are shown using Kruskal-Wallis and Dunn's Multiple Comparison Tests. (*C*) Venn diagram showing the shared and unique accurate genes across SCFM2-PAO1, SCFM2-Calprotectin and SCFM5-PAO1 models. Together, these 3 models capture the expression of all (4,909) but 259 *P. aeruginosa* PAO1 core genes (5.0%) during CF lung infection. (*D*) The average z-score for each gene in SCFM2-PAO1 and SCFM5-PAO1, demonstrating how the addition of metal-binding TPEN in SCFM2 alters the accuracy of PAO1 genes. Genes are considered accurate when their z-score is between −2 and 2. Genes are colored based on the conditions in which they are accurate, and annotations are shown for select genes. (*E*) AS_2 for TIGRFAM subcategories for *P. aeruginosa* CF clinical strains in SCFM5. The color in the middle represents the average AS_2 across individual replicates for all PAO1 genes (those with and without TIGRFAM designations). The next level out from the middle

of the circle contains TIGRFAM meta roles, the next contains TIGRFAM main roles, and the outer-most layer contains TIGRFAM sub roles. The area of each category is proportional to the number of genes in that category.

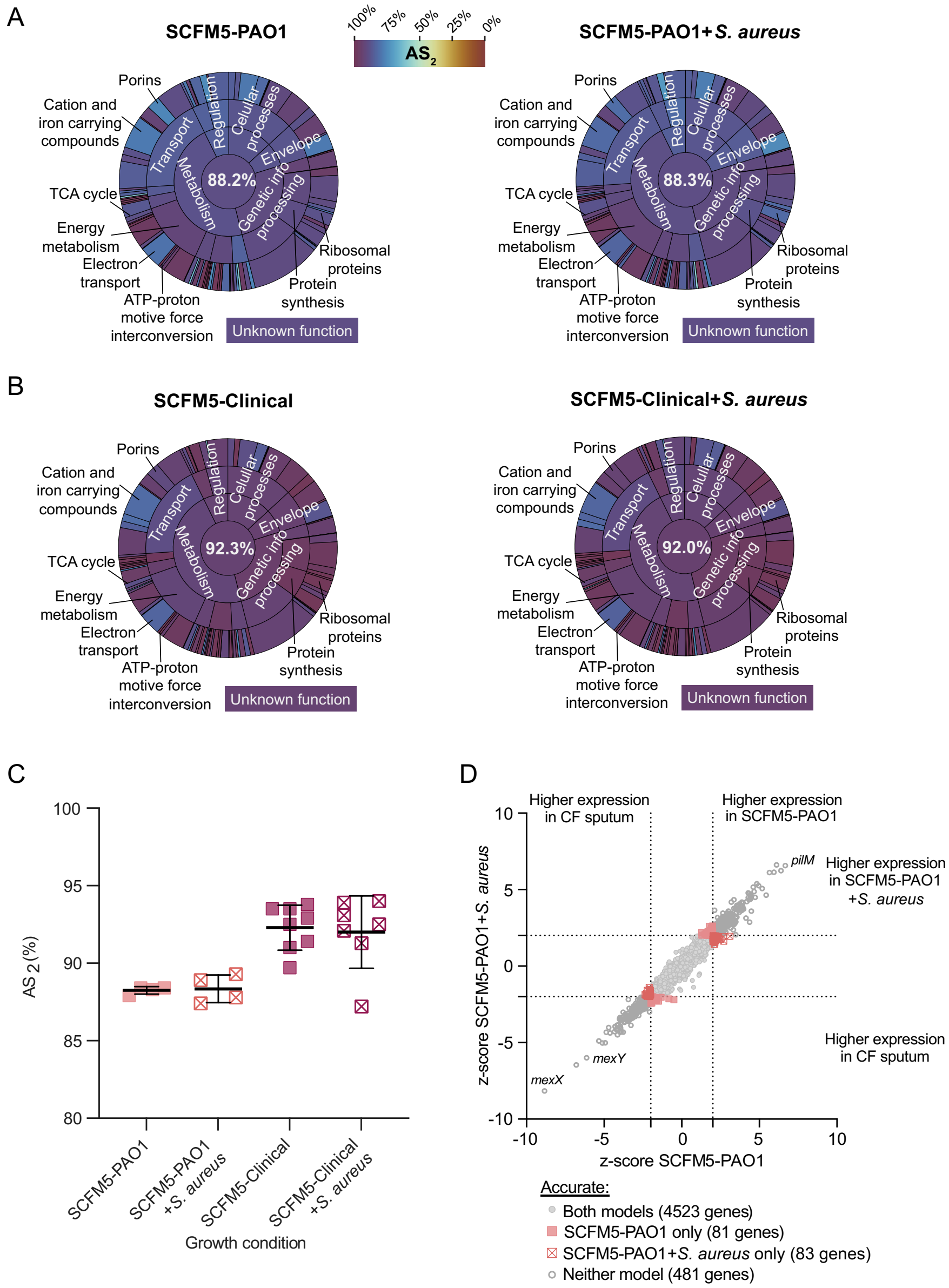

Fig. S6. Coculture with *S. aureus* has minimal impact on *P. aeruginosa* model accuracy. (*A* and *B*) AS_2 for TIGRFAM subcategories for *P. aeruginosa* PAO1 and CF clinical strains grown in the presence or absence of *S. aureus* in SCFM5. The color in the middle represents the average AS_2 across individual replicates for all PAO1 genes (those with and without TIGRFAM designations). The next level out from the middle of the circle contains TIGRFAM meta roles, the next contains TIGRFAM main roles, and the outer-most layer contains TIGRFAM sub roles. The area of each category is proportional to the number of genes in that category. (*C*) AS_2 for each replicate of each model. No significant differences were found after analysis with Kruskal-Wallis and Dunn's Multiple Comparison Tests. (*D*) The average z-score for each gene in SCFM5-PAO1 and SCFM5-PAO1+*S. aureus*, demonstrating that coculture of *P. aeruginosa* PAO1 with *S. aureus* has a negligible impact on gene expression accuracy. Genes are considered accurate when their z-score is between −2 and 2. Genes are colored based on the conditions in which they are accurate, and annotations are shown for select genes.

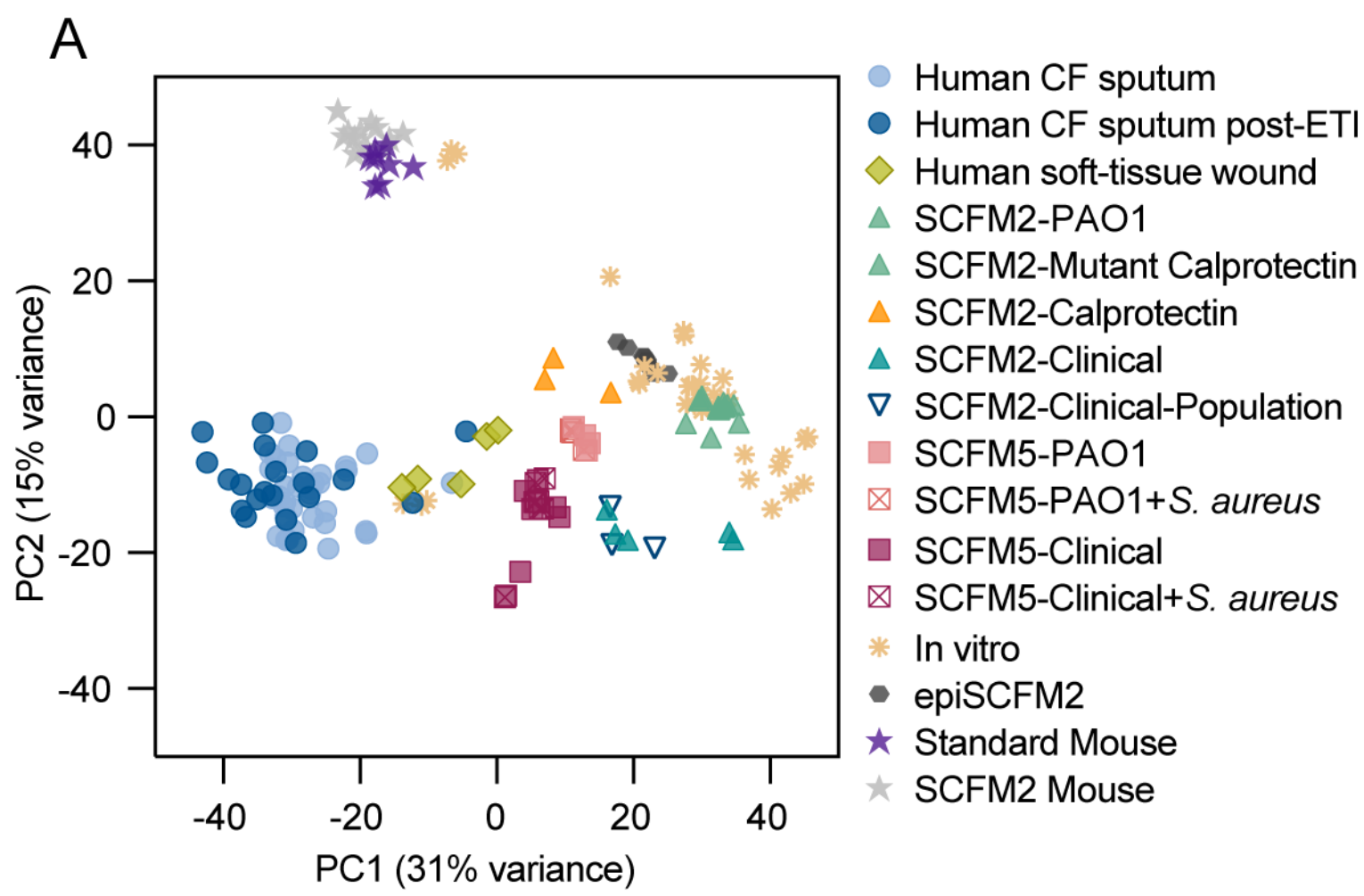

Fig. S7. Refined models remain accurate when benchmarked against post-ETI human sputum. Principal component analysis (PCA) of *P. aeruginosa* gene expression in pre- and post-ETI human CF sputum samples and preclinical model systems. The PCA was constructed using Variance Stabilizing Transformation (VST)-normalized read counts of the 500 genes (out of 5,168 core genes) with highest variability across samples using DESeq2. All samples and models included in this analysis are detailed in Dataset S1*A*.

Table S1. Demographic and clinical characteristics of the Dublin, Ireland cohort.

Characteristic	pre-ETI (n=10)	post-ETI Year 1 (n=5)	post-ETI Year 2 (n=5)
**Age (years), mean (SD)**	32.9 (5.6)	31.4 (4.7)	36.8 (7.0)
**Sex (% male)**	80%	60%	60%
**FEV_1 (% predicted), mean (SD)**	55.4 (17.8)	69.6 (17.4)	68.6 (18.9)
**FEV_1 (L), mean (SD)**	2.15 (0.73)	2.53 (0.8)	2.45 (0.81)
**Genotype group, n (%)**			
F508del/F508del	9 (90%)		
F508del/R560T	1 (10%)		
**Duration of chronic *P. aeruginosa* infection, mean (range)**	13 years (6-21 years)		

Table S2. Demographic and clinical characteristics of the Atlanta, Georgia cohort.

Characteristic	post-ETI Years 5-6 (n=10)
**Age (years), mean (SD)**	41.8 (11.5)
**Sex (% male)**	40%
**FEV_1 (% predicted), mean (SD)**	60.25 (19.8)
Pre-ETI FEV_1 (% predicted), mean (SD)	52 (23.6)
**FEV_1 (L), mean (SD)**	1.88 (0.50)
Pre-ETI FEV_1 (L), mean (SD)	1.66 (0.64)
**Genotype group, n (%)**	
F508del/F508del	4 (40%)
F508del heterozygous	4 (40%)
Other	2 (20%)
**Duration of chronic *P. aeruginosa* infection, mean (range)**	21 years (13-30 years)

Dataset S1: ***A.*** Sample metadata, mapping statistics, and counting statistics for all human sputum and in vitro samples in this study (1-8). ***B.*** Annotation of the *P. aeruginosa* PAO1 genome, including hand-curated TIGRFAM annotations used in this study and core genes. ***C.*** rlog-normalized count data for all RNA-seq samples, including the variance in expression across human sputum metatranscriptomes pre- and post-ETI. ***D.*** VST-normalized count data for all RNA-seq samples, including the variance in expression across human sputum metatranscriptomes. ***E.*** Raw count data for all RNA-seq samples.

Dataset S2: ***A.*** Accuracy of *P. aeruginosa* PAO1 genes in subgroups of three randomly chose post-ETI samples at a time compared to gene expression in the 34 benchmark pre-ETI sputum metatranscriptomes. ***B.*** Differential expression analysis of all 5,168 core genes in post-ETI versus pre-ETI transcriptomes. ***C.*** Differential expression analysis of all 5,168 core genes in post-ETI versus pre-ETI transcriptomes restricted to six Dublin patients.

Dataset S3: ***A.*** Accuracy of *P. aeruginosa* PAO1 genes in each replicate of each experimental model relative to pre-ETI human CF sputum transcriptomes. ***B.*** Accuracy and mean z-score of *P. aeruginosa* PAO1 genes across each experimental model. ***C***. Accuracy of *P. aeruginosa* PAO1 genes each experimental model relative to post-ETI human CF sputum transcriptomes.

References

1. A. Dotsch *et al.*, The Pseudomonas aeruginosa transcriptome in planktonic cultures and static biofilms using RNA sequencing. *PLoS One* **7**, e31092 (2012).
2. A. Dotsch *et al.*, The Pseudomonas aeruginosa Transcriptional Landscape Is Shaped by Environmental Heterogeneity and Genetic Variation. *mBio* **6**, e00749 (2015).
3. J. L. Murray, T. Kwon, E. M. Marcotte, M. Whiteley, Intrinsic Antimicrobial Resistance Determinants in the Superbug Pseudomonas aeruginosa. *mBio* **6**, e01603-01615 (2015).
4. D. M. Cornforth *et al.*, Pseudomonas aeruginosa transcriptome during human infection. *Proc Natl Acad Sci U S A* **115**, E5125-E5134 (2018).
5. D. M. Cornforth, F. L. Diggle, J. A. Melvin, J. M. Bomberger, M. Whiteley, Quantitative Framework for Model Evaluation in Microbiology Research Using Pseudomonas aeruginosa and Cystic Fibrosis Infection as a Test Case. *mBio* **11** (2020).
6. B. G. Fritz *et al.*, Transcriptomic fingerprint of bacterial infection in lower extremity ulcers. *APMIS* **130**, 524-534 (2022).
7. G. R. Lewin *et al.*, Application of a quantitative framework to improve the accuracy of a bacterial infection model. *Proc Natl Acad Sci U S A* **120**, e2221542120 (2023).
8. R. P. Duncan *et al.*, Improvement of a mouse infection model to capture Pseudomonas aeruginosa chronic physiology in cystic fibrosis. *Proc Natl Acad Sci U S A* **121**, e2406234121 (2024).
